# Fracture of the Superior Maxilla

**Published:** 1895-12

**Authors:** W. D. M. Mason

**Affiliations:** Fort Worth.


ARTICLE IV.

### FRACTURE OF THE SUPERIOR MAXILLA.

BY W. D. M. MASON, FORT WORTH.

Read at Houston, May, 1895.

May the 9th, 1894, Mrs. B., a comely widow of 38 summers, came to me with the following story, viz:

"Last night during a thunder and rain storm, while out in the back yard rounding up my children preparatory to putting them to bed, I was so blinded and shocked by the constant flashes of lightening, and before realizing what was going on was thrown violently to the ground. My protracted absence caused sister much uneasiness, and on going out in search of me, was horrified and frightened at finding me stretched out on the ground in the rain. With the assistance of our children I was carried into the house. The whole atmosphere was heavily charged with electricity, and the last thing I remembered everything was whirling around like a merry-go-around. In falling my mouth must have struck a scraper used in railroad building, completely knocking out one of my upper front teeth, which was picked up next morning near the scraper."

Upon examination, I found Mrs. B.'s story to be true as to the tooth being out; also fracture of the superior maxilla. She had the right central incisor in her hand bag, while the left one was merely hanging in the mouth. Resetting the bone and teeth, after thorough preparation, I wired from cuspid to cuspid with small copper wire, drawing the lingual and labial wire together between each tooth, making them immovable. Of course, Mr. President and members of the Texas Dental Association, it is unnecessary to go into details as to further treatment; suffice it to say that a thorough antiseptic treatment was kept up for four weeks, not a drop of pus forming. Removing the wires, I was gratified at the result of the job, perfect union having taken place. I might add that, during the whole time the wires were worn the patient suffered little if any pain or inconvenience, except the first few days, when there was considerable nausea. The occlusion is as perfect as before the accident, and but for the loss of a few pieces of alocali, the teeth are as firm as if nothing had happened. I see the patient frequently and am glad to state that she uses them with impunity, even to the biting of threads. I am at a loss, Mr. President, to account for Mrs. B.'s shock, for I remember very distinctly there was no sound of anything being struck by lightening at the hour she narrated, though the whole elements seemed to be a perfect spider web of lightning. I somehow doubted her veracity and began "joshing" her about attending an "Irish wake," when she reiterated it with even more emphasis.—*Texas Dental Jonrnal.*

